# Clinical Features and Predictive Risk Factors for Prognosis in Invasive Pulmonary Aspergillosis and Pulmonary Mucormycosis

**DOI:** 10.1111/crj.70186

**Published:** 2026-04-19

**Authors:** Yu Bai, Ming Wei, Jun Liu, Xi Chen, Li Gu, Yinqun Guo

**Affiliations:** ^1^ Department of Infectious Diseases and Clinical Microbiology, Beijing Institute of Respiratory Medicine and Beijing Chao‐Yang Hospital Capital Medical University Beijing China

**Keywords:** hemoglobin A1c, invasive pulmonary aspergillosis, neutrophil‐to‐lymphocyte ratio, predictive risk factors, pulmonary mucormycosis

## Abstract

**Background:**

Invasive pulmonary aspergillosis (IPA) and pulmonary mucormycosis (PM) stand as the most prevalent invasive mold pulmonary infections. The incidence of IPA and PM has progressively increased. Untimely or inappropriate intervention amplifies mortality rates in patients affected by IPA and PM. There exist numerous commonalities between the two with regard to the population susceptible to the disease and imaging characteristics. This renders it challenging to differentiate them in certain clinical practices, resulting in issues such as the inappropriate selection of treatment plans. Early and expeditious differential diagnosis of invasive pulmonary mold infections and prompt identification of severe cases are critical challenges in clinical practice.

**Methods:**

A retrospective cohort study encompassed IPA and PM patients admitted to Beijing Chao‐Yang Hospital from 2017 to 2022. Patients in the cohort were categorized into PM and IPA groups. A comprehensive analysis of clinical characteristics, laboratory parameters, and chest radiology findings was conducted. Subsequently, a comparative assessment of the prognosis between the two patient groups was carried out. All patients with invasive pulmonary mold infection were classified based on prognosis, and independent risk factors for poor prognosis were identified. Subsequent to these findings, exploration of novel disease assessment tools was undertaken, and their diagnostic efficacy was evaluated.

**Results:**

In comparison to IPA, PM patients exhibited a younger age profile, with a higher incidence of diabetes and solid organ transplantation. PM occurrences postinfluenza infection were less frequent than IPA. Radiologically, consolidation and bronchial lumen stenosis were more prevalent in PM patients. Additionally, the diagnosis of PM patients relied more on pathological confirmation. No significant disparities were noted regarding ICU stays, mechanical ventilation ratios, and 90‐day mortality between PM and IPA. Postinfluenza infection and the neutrophil‐to‐lymphocyte ratio (NLR) were identified as independent risk factors for ICU stays in PM/IPA patients. Postinfluenza infection and elevated hemoglobin A1c (HbA1c) levels were independent risk factors for mechanical ventilation. NLR, HbA1c levels, and postinfluenza infection collectively enhanced the predictive capacity of existing assessment tools for adverse outcomes in PM/IPA patients.

**Conclusions:**

PM patients exhibit distinctions from IPA in certain clinical characteristics, laboratory parameters, and chest radiology findings. Nevertheless, both PM and IPA patients experienced higher 90‐day mortality and ICU utilization. The combination of NLR and HbA1c with existing disease assessment tools proves effective in prognosticating the disease, particularly during influenza epidemic seasons.

## Graphical abstract

PM patients exhibit distinctions from IPA in certain clinical characteristics, laboratory parameters, and chest radiology findings. Nevertheless, both PM and IPA patients experienced higher 30‐day mortality and ICU utilization. The combination of NLR and HbA1c with existing disease assessment tools proves effective in prognosticating the disease, particularly during influenza epidemic seasons.

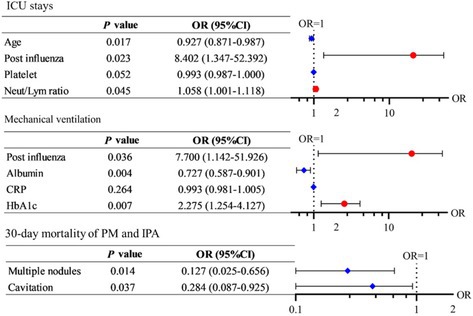

**Abbreviations**
APACHE‐II: Acute Physiology and Chronic Health Evaluation‐II
AUC: area under the curve
CT: computed tomography
CURB‐65: confusion, uremia, respiratory rate, blood pressure, age ≥ 65 years
HbA1c: hemoglobin A1c
IPA: invasive pulmonary aspergillosis
IQR: interquartile range
mNGS: metagenomic next‐generation sequencing
NLR: neutrophil‐to‐lymphocyte ratio
OR: odds ratio
PCR: polymerase chain reaction
PM: pulmonary mucormycosis
ROC: receiver operator characteristic
SD: standard deviation
SOFA: Sequential Organ Failure Assessment

## 1 Introduction

Invasive mold pulmonary infection represents a form of pulmonary fungal infection associated with heightened mortality and substantial morbidity. Among these infections, invasive pulmonary aspergillosis (IPA) and pulmonary mucormycosis (PM) emerge as the most prevalent. In recent years, there has been an unexpected surge in the incidence of invasive mold pulmonary infections. Global estimates indicate approximately 250 000 cases of invasive aspergillosis and over 10 000 cases of mucormycosis annually [1]. The escalating occurrences of solid organ transplantation, hematopoietic stem cell transplantation, immunotherapy, and viral infections [2], particularly COVID‐19, have been major contributors to the increased prevalence of invasive mold pulmonary infections. Recent studies on COVID‐19‐associated IPA have reported incidences ranging from 3% to 33% [3, 4], and it is suggested that the incidence of PM may be underestimated [5]. Prior research has identified female gender, advanced age (≥ 65 years), intubation, bone marrow transplantation, acute renal failure, other infectious diseases, and steroid use as predictive factors for mortality or adverse outcomes in IPA [6, 7, 8].

However, with the emergence of invasive fungal infections in nonimmunodeficient hosts and various types of immunodeficient hosts, the risk factors for poor prognosis may have undergone changes. It is crucial to note that swift identification of PM alongside IPA is essential as PM may necessitate distinct therapeutic regimens.

The objective of this study was to scrutinize the similarities and disparities in clinical manifestations between IPA and PM within a retrospective cohort of invasive mold pulmonary infections. Additionally, the study aimed to analyze potential predictors of poor prognosis in patients with diverse invasive mold pulmonary infections and using clinical decision tools to offer insights for the early identification of distinct infection etiologies and the recognition of critically ill patients in clinical practice.

## 2 Methods

### 2.1 Patient Population and Study Setting

We conducted a retrospective cohort study at Beijing Chao‐yang Hospital, Beijing, China, an academic center with 2500 beds divided into three sites. Adult patients (≥ 18 years) admitted to the departments of respiratory and critical care medicine, infectious diseases, and clinical microbiology between January 1, 2017, and December 31, 2022, with diagnoses of IPA or PM were reviewed for inclusion. Patients with IPA or PM who did not meet the corresponding diagnostic criteria and those with unknown prognoses were excluded.

The study received approval from the Institutional Review Board of Beijing Chao‐Yang Hospital (2017‐KE‐193). All methods were performed in accordance with the relevant guidelines and regulations as stipulated in the Declaration of Helsinki.

### 2.2 Data Sources and Definitions

Data were obtained from a database of electronic health records encompassing all patients admitted to Beijing Chao‐Yang Hospital between January 2017 and December 2022. This dataset included demographics, past medical history, chest computerized tomography (CT) reports, laboratory findings, vital signs, microbiology results, intensive care data, and outcomes upon discharge. Demographic information encompassed gender and age. Regarding vital signs and laboratory parameters, the initial measurements taken upon admission were used. Radiology reports from chest CT scans conducted within the first 48 h of admission or 48 h before hospitalization were independently reviewed by two radiologists for five signs of IPA/PM, including consolidation, cavitation, multiple nodules, bronchial lumen stenosis, and pleural effusion. Microbiological analyses conducted 24 h before to 48 h after admission were collected. Smears, cultures, polymerase chain reaction (PCR), or metagenomic next‐generation sequencing (mNGS) from nasopharyngeal samples, lower respiratory tract (LRT: sputum and bronchoalveolar lavage fluid), and blood were tested.

CURB‐65 (confusion, uremia, respiratory rate, blood pressure, age ≥ 65 years) [9], SOFA (Sequential Organ Failure Assessment) [10], APACHE (Acute Physiology and Chronic Health Evaluation)‐II [11] scores were evaluated within 48 h of admission. The specific evaluation methods of these three assessment tools refer to the corresponding evaluation criteria.

### 2.3 Diagnostic Criteria

IPA: We referenced the guidelines established by the European Organization for Research and Treatment of Cancer and Mycoses Study Group Education and Research Consortium (EORTC‐MSGERC) that defined the diagnosis of possible, probable, and proven IPA in patients who have underlying conditions [12]. Patients demonstrating evidence of invasion (positive histopathology, direct exam, or cultures in sterile sites with evidence of infection) were categorized as proven IPA. Those meeting host criteria, clinical criteria, and mycological criteria simultaneously were classified as probable IPA [13].

PM: The diagnosis of patients with PM was made with reference to relevant literature. Identification of Mucor fungi in lung tissue samples through etiology or histopathology can be a core diagnostic basis. Clinical diagnosis is based on host factors and clinical manifestations, such as characteristic imaging findings in the lungs. Microbiological evidence, including sputum, bronchoalveolar lavage fluid, and bronchial brush extract, can be used for etiological diagnosis to identify Mucor fungi [14, 15].

### 2.4 Outcomes

The primary outcome was 90‐day mortality after the diagnosis was confirmed, differentiated between patients with IPA and PM. Secondary outcomes included the incidence of ICU usage and the incidence of mechanical ventilation. We also evaluated the efficacy of prognostic scores (CURB‐65, SOFA, and APACHE‐II). The last follow‐up date for survival status was the date of 90‐day after the diagnosis was confirmed.

### 2.5 Statistical Analysis

Statistical analyses were conducted using SPSS Statistics V.23 (IBM Inc., Chicago, Illinois, USA) and MedCalc V.19.6.4 (MedCalc Software, Ostend, Belgium). Data were presented as either the median with interquartile range (IQR) or mean ± standard deviation (SD) for numerical variables, count, and percentage for categorical variables where appropriate. *T*‐tests were employed for continuous variables with normal distribution, whereas Mann–Whitney U tests were used for those with nonnormal distribution. Chi‐square tests were utilized for comparing categorical variables. Variables that exhibited significant differences between different outcomes were considered potential risk factors. Logistic regression analysis was performed to identify predictive factors for different outcomes of all patients/IPA/PM. The results were presented as estimates of relative risk expressed as a relative risk (RR) with a 95% confidence interval (CI). Statistical significance was set at *p* < 0.05. Receiver operating characteristic (ROC) curves were plotted to calculate the area under the curve (AUC) for different disease prediction tools. The logistic regression model for combination diagnoses was established based on the findings. The prediction probabilities of the combinations were calculated, the ROC curve was plotted according to the predictive probability, and then, the AUC for different combination diagnoses was calculated.

## 3 Results

### 3.1 Demographic Characteristics, Underlying Diseases, and Clinical Manifestations of Patients With Different Types of Invasive Mold Pulmonary Infection

**FIGURE 1:**
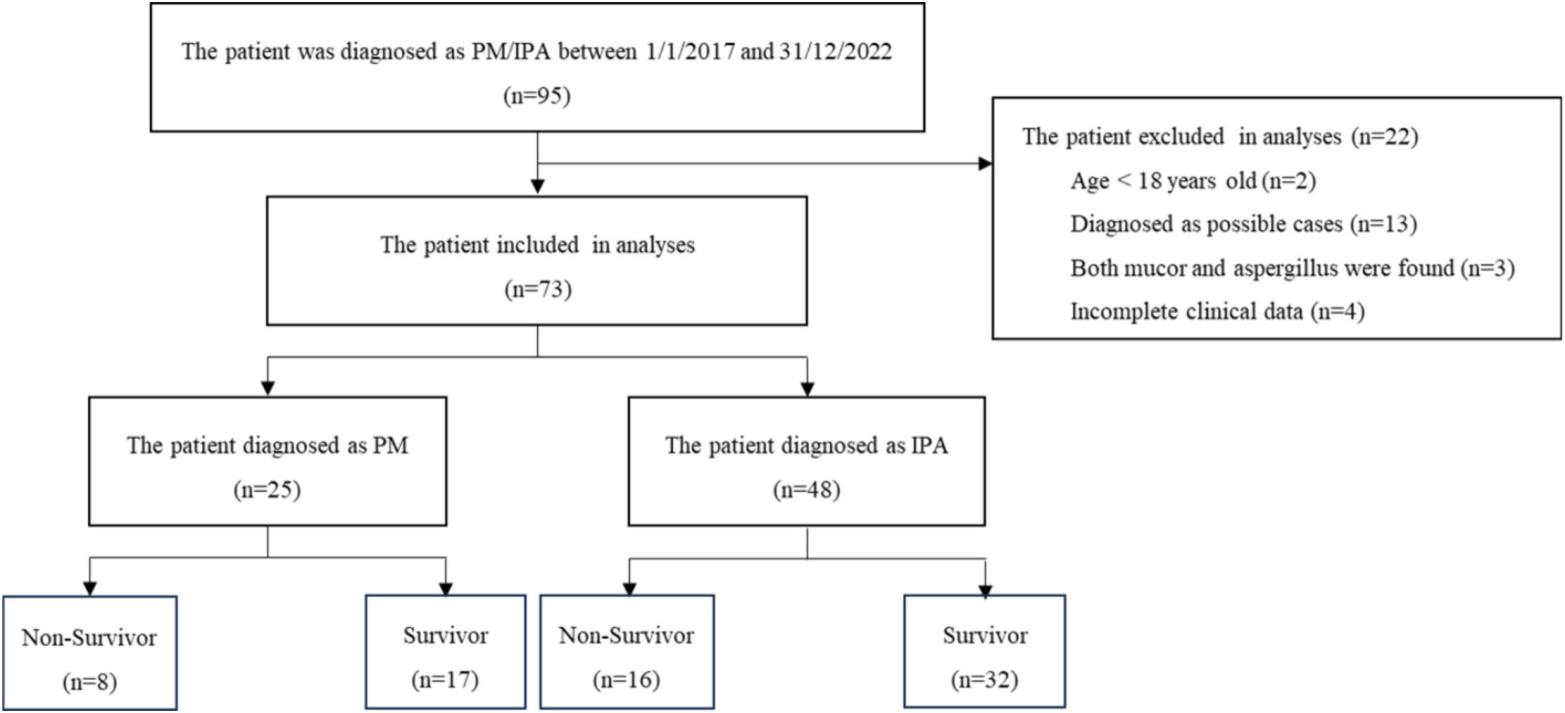
Study flowchart.

Between 1 January 2017 and 31 December 2022, 95 patients were diagnosed with PM or IPA. Following the exclusion of 22 patients, 73 were included in the final analysis. The group diagnosed with PM comprised 25 patients, and the IPA group comprised 48 patients. The flowchart is depicted in Figure 1.

The demographic characteristics, clinical manifestations, laboratory findings, and chest radiology results of patients in the two groups are presented in Table 1. Patients hospitalized for PM were younger than IPA patients (*p* = 0.005). Diabetes mellitus and solid organ transplant recipients were more prevalent in PM patients (*p* < 0.001 and *p* = 0.034, respectively). Concurrently, more PM patients had previously received antifungal agents (*p* < 0.001). In contrast, the incidence of IPA following influenza was significantly higher than that of PM (*p* = 0.016). In laboratory data, neutrophil‐to‐lymphocyte ratio (NLR), CRP, and GM test in serum were higher in IPA patients (*p* = 0.047, 0.024, and 0.002). The predominant Mucor fungi in patients with PM were *Rhizopus arrhizus*, followed by *Rhizomucor pusillus* and *Rhizopus microsporus*. In patients with IPA, *Aspergillus fumigatus* was the most prevalent, followed by *Aspergillus flavus* and *Aspergillus nidulans*. Regarding chest CT findings, consolidation and bronchial lumen stenosis were more frequent in PM patients (*p* = 0.018 and 0.008). Additionally, a higher proportion of PM patients received a proven diagnosis, whereas a probable diagnosis was more common in IPA (*p* < 0.001). No significant differences were noted in other indices between the two groups.

**TABLE 1:** Comparison of clinical characteristics, laboratory, and chest radiology findings between IPA and PM.

	PM (*n* = 25)	IPA (*n* = 48)	*p*
Ages, years	52.64 ± 13.74	61.83 ± 12.25	0.005R
Sex, male, no. (%)	15 (60.0)	39 (81.3)	0.050
Underlying disease, no. (%)			
DM	20 (80.0)	16 (33.3)	< 0.001
DKA	3 (12.0)	3 (6.3)	0.407
Chronic lung disease	5 (20.0)	12 (25.0)	0.628
Solid organ cancer	0	4 (8.3)	0.292
Hematologic disease	1 (4.0)	4 (8.3)	0.655
Solid organ transplantation	5 (20.0)	2 (4.2)	0.034
Autoimmune disease	0	5 (10.4)	0.158
Hepatopathy	3 (12.0)	3 (6.3)	0.407
Chronic kidney disease	4 (16.0)	6 (12.5)	0.683
Systemic steroid, no. (%)	13 (52.0)	21 (43.6)	0.503
Immunosuppressant, no. (%)	6 (24.0)	10 (20.8)	0.756
Postinfluenza, no. (%)	1/18 (5.6)	12/37 (32.4)	0.016
Post‐COVID‐19, no. (%)	4/21 (19.0)	8/27 (29.6)	0.397
Prior antifungal agent,^a^ no. (%)	21 (84.0)	16 (33.3)	< 0.001
Breakthrough fungal infection, no. (%)	8 (32.0)	0	< 0.001
Blood routine test			
WBC, ×10^9^/L	7.80 (6.31, 14.78)	11.19 (7.72, 15.89)	0.189
N, ×10^9^/L	5.47 (4.13, 12.53)	9.17 (5.13, 13.42)	0.216
L, ×10^9^/L	1.34 ± 0.81	1.12 ± 0.91	0.333
NLR	3.47 (2.71, 8.88)	9.86 (5.60, 21.03)	0.047
Hgb, g/L	103.08 ± 27.21	108.19 ± 25.03	0.426
PLT, ×10^9^/L	243.00 (135.50, 343.00)	220.50 (112.25, 337.00)	0.474
Inflammation‐related test			
ESR, mm/h	27.00 (13.00, 49.50)	35.50 (20.50, 64.00)	0.278
CRP, mg/L	5.67 (1.79, 11.05)	10.96 (4.84, 22.65)	0.024
CD4^+^ T cells, /μL	311.00 (129.50, 682.00)	241.00 (86.00, 521.00)	0.334
Microbiological tests			
G test	10.00 (10.00, 95.03)	37.45 (10.00, 206.66)	0.157
GM test (serum)	0.25 (0.14, 0.65)	0.57 (0.28, 1.55)	0.002
GM test (balf)	—	3.11 (1.11, 4.74)	
PCT, ng/mL, mean ± SD	0.11 (0.05, 1.58)	0.18 (0.07, 1.11)	0.501
HbA1C, %	7.30 (5.80, 8.30)	6.50 (6.00, 8.15)	0.162
Chest CT findings			
Consolidation, no. (%)	21 (84.0)	27 (56.3)	0.018
Cavitation, no. (%)	13 (52.0)	23 (47.9)	0.741
Multiple nodules, no. (%)	11 (44.0)	11 (22.9)	0.062
Bronchial lumen stenosis, no. (%)	9 (36.0)	5 (25.0)	0.008
Pleural effusion, no. (%)	13 (52.0)	20 (41.7)	0.400
Diagnostic classification, no. (%)			
Proven, no. (%)	13 (52.0)	1 (2.1)	< 0.001
Probable, no. (%)	12 (48.0)	47 (97.9)	

^a^ Patients who have been treated with antifungal agents within the past 6 months.

### 3.2 The Outcomes of IPA and PM Patients

The 90‐day mortality was 32.0% in PM patients compared with 33.3% in IPA patients in the cohorts. In‐hospital durations, percentages of ICU admissions, and mechanical ventilation were similar between the two groups. These prognostic markers were observed in PM and IPA patients, respectively. For both PM and IPA patients, the ICU utilization rate and mechanical ventilation rate of nonsurvivor patients were higher than those of surviving patients (as shown in Table 2).

**TABLE 2:** Comparison of different adverse prognostic events in all patients.

	PM				IPA				
	All	Survivors	Nonsurvivors	*p*	All	Survivors	Nonsurvivors	*p*	*p*
Hospital stays, days	15.00 (10.50, 21.50)	17.00 (13.50, 29.00)	10.00 (1.75, 14.00)	0.003	18.00 (9.25, 23.00)	18.00 (11.25, 23.00)	18.00 (8.00, 25.00)	0.630	0.500
ICU stays, no. (%)	9 (36.0)	4 (23.5)	5 (62.5)	0.060	18 (37.5)	9 (28.1)	9 (56.3)	0.058	0.900
Mechanical ventilation, no. (%)	8 (32.0)	4 (23.5)	4 (50.0)	0.192	17 (35.4)	6 (18.8)	11 (68.8)	0.001	0.770
90‐Day mortality of invasive mold pneumonia, no. (%)		8 (32.0)				16 (33.3)			0.908

### 3.3 Predictive Risk Factors for Prognosis in PM and IPA

Relevant indicators that exhibited statistical significance in the univariate analysis were incorporated into the logistic regression model. The indicators are presented in Tables S1–S3. In PM patients, the use of immunosuppressants (odds ratio [OR] = 23.333, 95% CI 1.948–279.429, *p* = 0.013) was identified as the predictive risk factor for ICU stays in PM patients. An increase in PCT (OR = 6.383, 95% CI 1.026–39.322, *p* = 0.046) was identified as the predictive risk factor for mechanical ventilation. Solid organ transplantation (OR = 16.000, 95% CI 1.381–184.405, *p* = 0.027) was identified as the predictive risk factor for 90‐day mortality in PM patients.

In IPA patients, postinfluenza infection (OR = 6.760, 95% CI 1.156–39.538, *p* = 0.034) and an increase in the absolute number of neutrophils (OR = 1.129, 95% CI 1.001–1.273, *p* = 0.049) were identified as predictive risk factors for ICU stays. Diabetes mellitus (OR = 6.945, 95% CI 1.568–30.764, *p* = 0.011) and elevated CRP levels (OR = 1.080, 95% CI 1.008–1.156, *p* = 0.029) were determined as predictive risk factors for mechanical ventilation.

Simultaneously, elevated CRP levels (OR = 1.100, 95% CI 1.025–1.180, *p* = 0.008) were also considered a predictive risk factor for 90‐day mortality in IPA patients.

**FIGURE 2:**
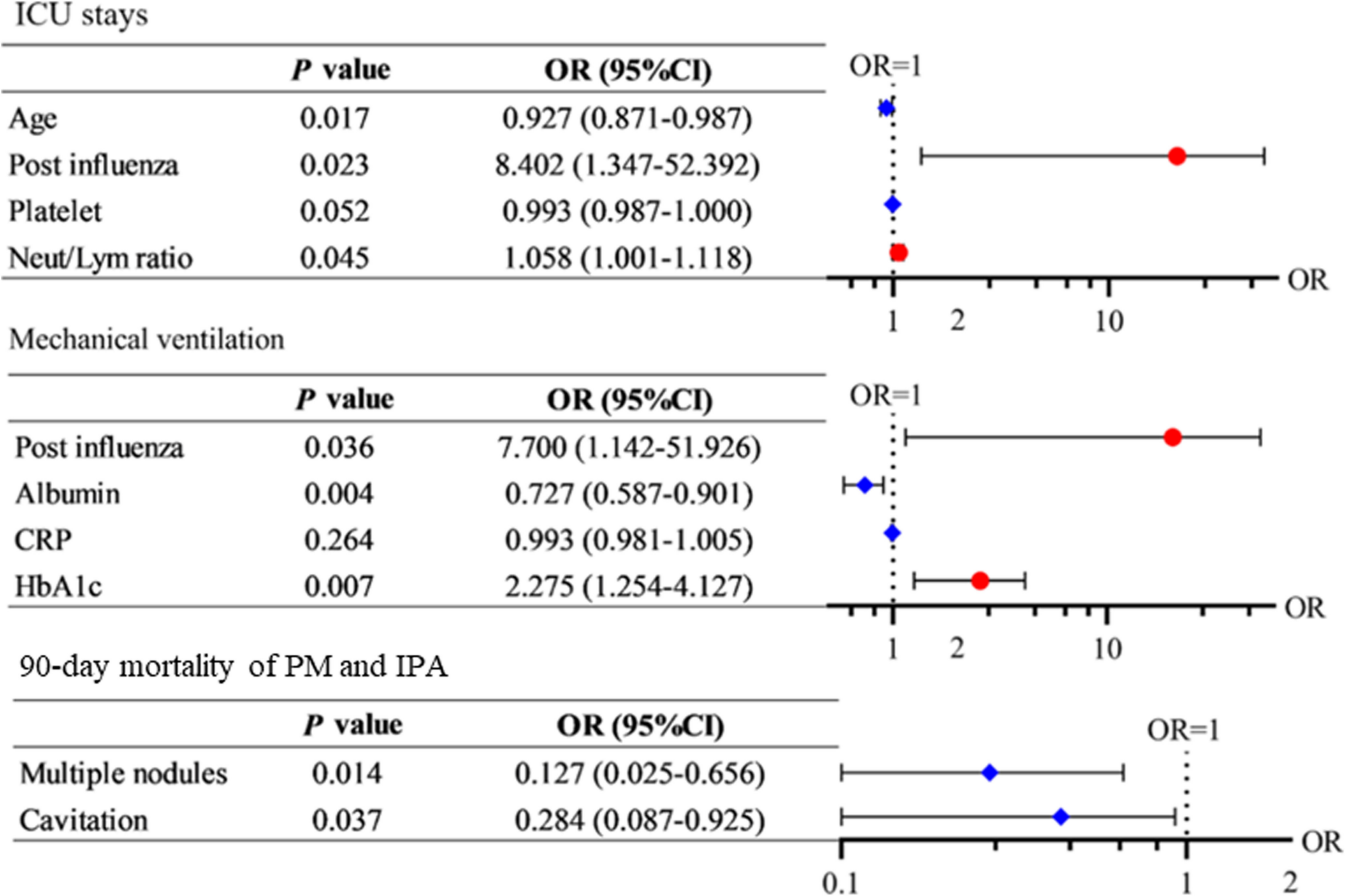
Risks for different outcomes for PM and IPA patients. The OR and 95% CIs were plotted, with red and blue representing OR > 1 and OR < 1, respectively.

Subsequently, we pooled PM and IPA patients for analysis; the results are shown in Figure 2. For all patients, postinfluenza infection (OR = 8.402, 95% CI 1.347–52.492, *p* = 0.023) and an elevated NLR (OR = 1.058, 95% CI 1.001–1.118, *p* = 0.045) were identified as predictive risk factors for ICU stays. Additionally, postinfluenza infection (OR = 7.700, 95% CI 1.142–51.926, *p* = 0.036) and increased HbA1c (OR = 2.275, 95% CI 1.254–4.127, *p* = 0.007) were also determined as predictive risk factors for mechanical ventilation. No meaningful findings were observed for predictive risk factors of 90‐day mortality.

### 3.4 Predictive Value of Severity Assessment Tools Compared With New Predictive Risk Factors for Prognoses of Patients With PM/IPA

**FIGURE 3:**
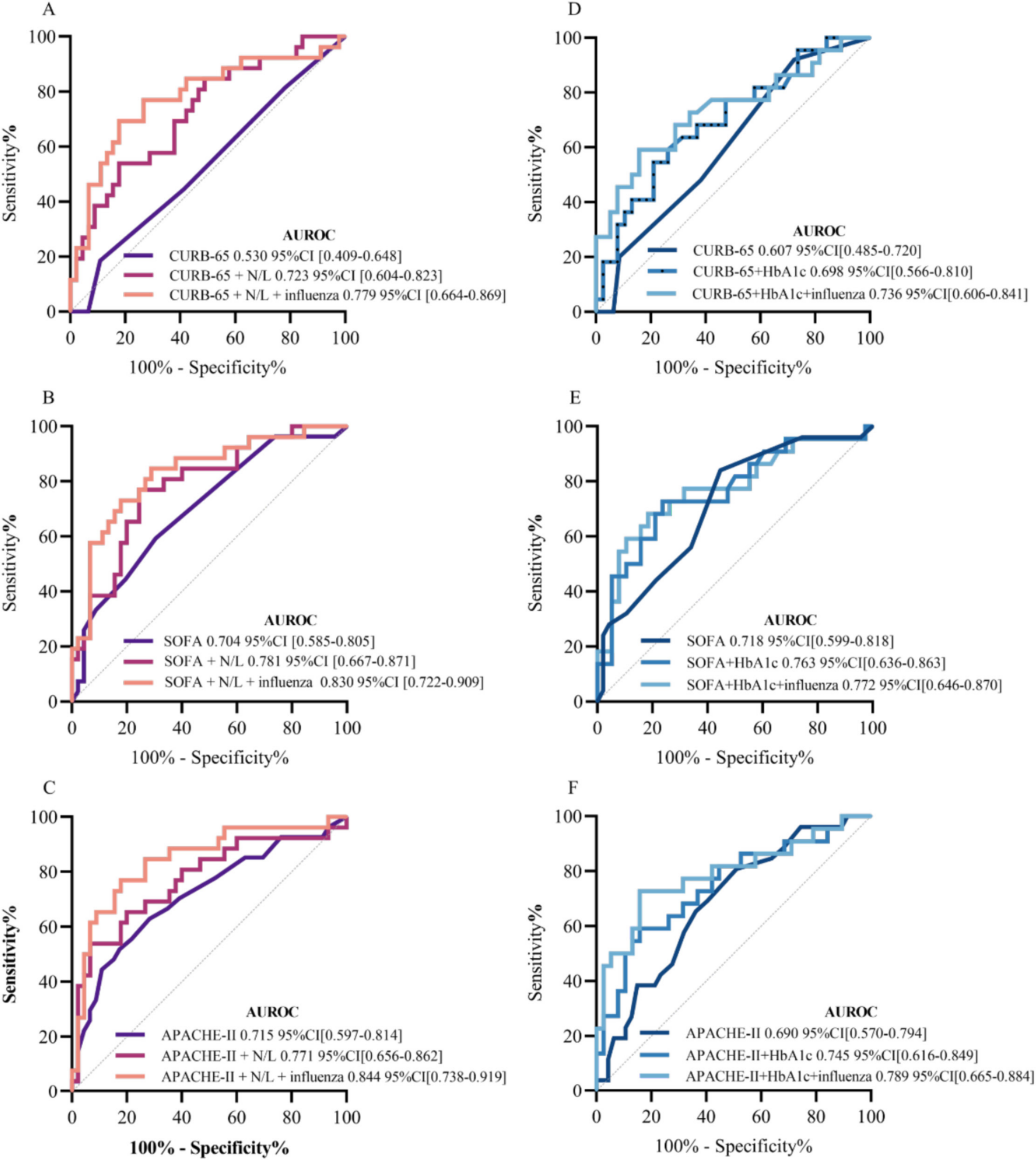
AUC of different assessment tools and different tools combined new predictors for different outcomes. The ROC curves were depicted in different colors. Figure 3A–C shows the outcomes of ICU stays; Figure 3D–F shows the outcomes of mechanical ventilation.

CURB‐65, SOFA, and APACHE‐II assessments were conducted for all patients with PM/IPA included in this study. The predictive effect of risk factors for different tools was assessed. The ROC curves are presented in Figure 3, and relevant data are provided in Table S4. Regarding the assessment tools for ICU stays, the findings indicated that the AUC of ROC for postinfluenza + NLR was 0.779 (95% CI 0.665–0.869, *p* < 0.001), with a sensitivity of 69.23% and specificity of 82.22%. Compared with CURB‐65, postinfluenza + NLR demonstrated higher predictive accuracy (*p* = 0.002). In comparison with SOFA and APACHE‐II, postinfluenza + NLR improved prediction accuracy, although the differences were not statistically significant (*p* = 0.396 and 0.483).

Regarding the assessment tools for mechanical ventilation, the AUC of ROC for postinfluenza + HbA1c was 0.709 (95% CI 0.577–0.819, *p* < 0.005), with a sensitivity of 72.73% and specificity of 71.05%. Postinfluenza + HbA1c improved prediction accuracy compared with CURB‐65, SOFA, and APACHE‐II, although the differences were not statistically significant (*p* = 0.317, 0.769, and 0.751).

Regarding the assessment tools for 90‐day mortality, the AUC of ROC for CURB‐65, SOFA, and APACHE‐II were 0.702, 0.629, and 0.602, respectively. No appropriate predictors were identified to further increase the AUC of ROC.

## 4 Discussion

In this retrospective cohort study, we meticulously investigated adult patients admitted to the hospital with PM or IPA. The key findings were as follows: (1) In comparison to IPA, PM patients were of a younger age. Among underlying diseases, diabetes and solid organ transplantation were more prevalent in PM patients. However, PM following influenza infection was less common than IPA. Radiologically, consolidation and bronchial lumen stenosis were more frequently observed in PM patients. Additionally, the diagnosis of PM relied more on pathological assessments, whereas clinical diagnosis was more common in the diagnostic process of IPA. (2) No significant differences were observed concerning ICU stays, mechanical ventilation ratio, or 90‐day mortality between PM and IPA. However, both PM and IPA exhibited ICU stays, mechanical ventilation ratios, and 90‐day mortality rates exceeding 30%. (3) Postinfluenza infection and NLR were identified as independent risk factors for ICU stays in PM/IPA patients. Postinfluenza infection and elevated HbA1c levels were determined as independent risk factors for mechanical ventilation. (4) NLR, HbA1c levels, and postinfluenza infection contribute to enhancing the predictive capacity of existing assessment tools for adverse outcomes in PM/IPA patients.

Invasive pulmonary mold infections represent highly perilous diseases. Particularly, the case fatality rate of PM/IPA without proper treatment is substantial. Among patients with hematologic malignancies, invasive aspergillosis stands out as the most prevalent type of invasive fungal disease, succeeded by Candida and Mucor [16]. In severely ill patients, Aspergillus and Mucor rank as the second most common fungal pathogens [17]. Although clinicians have progressed in their comprehension of invasive mold infections and can now identify them at earlier stages, the identification of fungal species remains inadequate, potentially causing delays in the treatment of PM patients. Previous studies have inadequately addressed the distinctions between PM and IPA, and our study aims to fill this knowledge gap to some extent.

Globally, mucormycosis carries a substantial disease burden, and its incidence is on the rise. The estimated global incidence of mucormycosis varies from 0.005 to 1.7 per 1 million people [18]. Europe bears the highest disease burden of mucormycosis, whereas India accounts for the largest number of mucormycosis patients [19]. Previous studies have not revealed clear underlying disease differences between PM and IPA [20]. However, our study identified diabetes and solid organ transplantation as more prevalent in PM. Among diabetic patients, Mucor is more prone to invading pulmonary epithelial cells and vascular endothelial cells, leading to local thrombosis formation in invaded blood vessels and subsequent tissue ischemic necrosis. The hyperglycemic environment diminishes the adhesion and phagocytosis of macrophages, and Mucor infection can induce macrophage apoptosis, rendering diabetic patients more susceptible to pulmonary Mucor infection from multiple perspectives [21]. Conversely, solid organ transplant recipients undergoing prolonged treatment with glucocorticoids and immunosuppressants face an increased risk of macrophage/neutrophil dysfunction and secondary hyperglycemia, predisposing factors for PM [22]. Although PM post‐COVID‐19 has been widely reported [19], our results did not reveal any particular significance in PM post‐COVID‐19, possibly because of the limited number of COVID‐19 cases included in this study. However, our findings also indicated that influenza infection predisposes individuals to IPA, aligning with prior research reports [20]. Part of the findings of this study seem to indicate a poor prognosis in younger patients. However, this result may be due to the fact that younger patients with the disease may have stronger host factors associated with immunosuppression. At the same time, young patients tend to be more aggressive in invasive treatment, so as to achieve such results.

In terms of laboratory tests, the GM test is crucial for distinguishing IPA and PM, with previous studies suggesting that this distinction is more pronounced in patients without hematological diseases [23, 24]. In our study, given the scarcity of patients with underlying hematological malignancies, the serum GM test of IPA patients was notably higher than that of PM patients. Additionally, CRP levels in IPA patients exceeded those in PM patients, indicating a potentially stronger systemic inflammatory response in IPA patients.

The radiological manifestations of PM are diverse and often nonspecific. Peribronchial ground‐glass opacity and multiple nodules can occur at different stages of PM [25]. Previous studies have reported radiological manifestations of the reversed halo sign in 19% to 94% of PM patients, with a higher prevalence in those with neutrophil deficiency [26]. Because of inconsistencies in the timing of patients undergoing chest imaging, the imaging characteristics vary. In our study, compared to IPA, PM patients exhibited more frequent imaging findings of consolidation and bronchial lumen stenosis. The angioinvasive nature of the disease contributes to the common occurrence of pulmonary necrosis in PM and may be the reason [25].

ICU stays, mechanical ventilation rates, and 90‐day mortality were elevated for both PM and IPA. Consequently, in addition to promptly establishing the direction of diagnosis for initiating appropriate antifungal therapy, it is crucial to identify patients at risk of severe illness at an earlier stage. Currently utilized disease assessment tools, including CURB‐65, SOFA, and APACHE‐II, exhibit limited performance in predicting a poor prognosis in PM and IPA patients.

In numerous infectious diseases, the circulating neutrophil count gradually increases, and neutrophil extracellular traps (NETs) exacerbate inflammation [27]. Neutrophils and lymphocytes are pivotal cellular components of the human host's defence system against infection. Circulatory lymphocytopenia has been documented in many patients with infectious diseases, particularly those in severe conditions [28]. Consequently, NLR has gained widespread use as a predictor of infectious diseases and other inflammation‐related conditions [29, 30, 31, 32]. In this study, NLR enhanced the predictive capability of existing disease assessment tools for ICU admission in IPA/PM patients. This proves valuable in forecasting the disease trajectory for patients admitted with IPA/PM.

HbA1c is commonly employed to monitor blood glucose control in patients. In certain diseases, HbA1c has also been identified as an independent risk factor for adverse outcomes such as death [33, 34, 35]. In our study, elevated HbA1c augmented the predictive efficacy of the disease assessment tool for the occurrence of mechanical ventilation. This suggests that the underlying disease in these patients is poorly controlled, leading to a more rapid progression of pulmonary infection. Nevertheless, the decision to perform mechanical ventilation is influenced by various circumstances beyond disease factors, potentially impacting its predictive accuracy compared to other prognostic indicators.

In our study, outcomes for IPA and PM following influenza infection were unfavourable. Postinfluenza infection increased the risk of both ICU stays and mechanical ventilation in these patients. Although we acknowledge that influenza pneumonia itself may exhibit rapid progression, leading to conditions such as ARDS, within the IPA/PM cohort, these patients, secondary to influenza, were more frequently admitted to the ICU and required mechanical ventilation. Nonetheless, this underscores the need to heighten our attention to IPA/PM patients during the influenza season.

This study presents certain limitations. Firstly, it is a retrospective analysis. In this retrospective cohort, some baseline data, particularly scores from disease assessment tools, could not be comprehensively reviewed, notably GCS scores, given the variability in recording by different physicians. Secondly, because of the infrequency of invasive mold infections, the sample size for this study remained modest. Simultaneously, the composition of admitted patients influenced by the disease spectrum resulted in fewer instances of hematological malignancies within this retrospective cohort. Presently, a multicenter study cohort on invasive fungal infections is underway, with the expectation that enhanced insights can be gleaned through the exploration of improved methods for identifying PM/IPA patients and validating the novel joint prediction tool established in this study.

In summary, PM patients exhibited a younger age, with diabetes and solid organ transplant recipients being more prevalent. PM subsequent to influenza infection was less common than IPA. Consolidation and bronchial lumen stenosis were more prevalent in PM patients. The diagnosis of PM patients relied more on pathological evaluation. Both PM and IPA cohorts demonstrated ICU stays, mechanical ventilation ratios, and 90‐day mortality rates exceeding 30%. Postinfluenza infection and NLR were identified as independent risk factors for ICU stays in PM/IPA patients. Postinfluenza infection and elevated HbA1c levels were independent risk factors for mechanical ventilation. NLR, HbA1c levels, and postinfluenza infection contributed to enhancing the predictive efficacy of existing assessment tools for adverse outcomes in PM/IPA patients.

## Author Contributions

Li Gu and Yiqun Guo play a guiding role in designing the study. Yu Bai mainly takes charge of statistical analysis of all data and writing of the manuscript. Yiqun Guo also performed original data collection. Jun Liu, Ming Wei, and Xi Chen were responsible for data analysis and recruiting the patients. All authors read and approved the final manuscript.

## Funding

The authors declare no conflicts of interest.

## Ethics Statement

The study was approved by the Institutional Review Board of Beijing Chao‐Yang Hospital (2017‐KE‐193). All methods in this study were carried out in accordance with relevant guidelines and regulations.

## Consent

As the anonymized data in the current retrospective study were used, a waiver of informed consent was included in the approval from the Institutional Review Board of Beijing Chao‐Yang Hospital.

## Conflicts of Interest

The authors declare no conflicts of interest.

## Supporting information


**Table S1:** Comparison of clinical characteristics, laboratory, and chest radiology findings between survivors and nonsurvivors in PM and IPA.
**Table S2:** Comparison of clinical characteristics, laboratory, and chest radiology findings between ICU patients and non‐ICU patients in PM and IPA.
**Table S3:** Comparison of clinical characteristics, laboratory, and chest radiology findings between mechanically ventilated patients and nonmechanically ventilated patients in PM and IPA.
**Table S4:** Predictive values of different assessment tools in different outcomes.

## Data Availability

The datasets are available from the corresponding author on reasonable request.
