# Supplementary material for: Clinical Features and Predictive Risk Factors for Prognosis in Invasive Pulmonary Aspergillosis and Pulmonary Mucormycosis
Source: Clin Respir J. 2026 Apr 19;20(4):e70186. doi: 10.1111/crj.70186 (PMC13092352; doi:10.1111/crj.70186)
Supplement: Supplementary file 1 — Table S1: Comparison of clinical characteristics, laboratory, and chest radiology findings between survivors and nonsurvivors in PM and IPA. Table S2: Comparison of clinical characteristics, laboratory, and chest radiology findings between ICU patients and non‐ICU patients in PM and IPA. Table S3: Comparison of clinical characteristics, laboratory, and chest radiology findings between mechanically ventilated patients and nonmechanically ventilated patients in PM and IPA. Table S4: Predictive values of different assessment tools in different outcomes. [file CRJ-20-e70186-s001.docx]

| **Supplementary Table 1** Comparison of clinical characteristics, laboratory and chest radiology findings between survivors and non-survivors in PM and IPA. | | | | | | | | | | |
| --- | --- | --- | --- | --- | --- | --- | --- | --- | --- | --- |
|  | PM (n=25) | | | IPA (n=48) | | | | All patients (n=73) | | |
|  | Survivor  (n=17) | Non-Survivor  (n=8) | P value | Survivor  (n=32) | | Non-Survivor  (n=16) | P value | Survivor  (n=49) | Non-Survivor  (n=24) | P value |
| Ages, years, mean±SD | 51.18±12.96 | 55.75±15.74 | 0.449 | 60.44±12.88 | | 64.63±10.73 | 0.269 | 57.22±13.53 | 61.67±12.99 | 0.186 |
| Sex, male, no. (%) | 12 | 3 | 0.116 | 25 | | 14 | 0.420 | 37 | 17 | 0.669 |
| Underlying disease, no. | | | | | | | | | | |
| DM | 13 | 7 | 0.506 | 10 | | 6 | 0.665 | 23 | 13 | 0.562 |
| DKA | 3 | 0 | 0.527 | 1 | | 2 | 0.223 | 4 | 2 | 0.980 |
| Chronic lung disease | 4 | 1 | 0.506 | 6 | | 6 | 0.157 | 10 | 7 | 0.406 |
| Solid organ cancer | - | - | - | 1 | | 3 | 0.074 | 1 | 3 | 0.075 |
| Hematologic disease | 1 | 0 | 1.000 | 3 | | 1 | 0.706 | 4 | 1 | 0.508 |
| Solid organ transplantation | 1 | 4 | 0.012 | 2 | | 0 | 0.546 | 3 | 4 | 0.165 |
| Autoimmune disease | - | - | - | 4 | | 1 | 0.487 | 4 | 1 | 0.508 |
| Hepatopathy | 1 | 2 | 0.187 | 3 | | 0 | 0.541 | 4 | 2 | 0.980 |
| Chronic kidney disease | 1 | 3 | 0.051 | 5 | | 1 | 0.329 | 6 | 4 | 0.611 |
| Systemic steroid, no. | 8 | 5 | 0.469 | 16 | | 6 | 0.537 | 23 | 11 | 0.929 |
| Immunosuppressant, no. | 2 | 4 | 0.042 | 7 | | 3 | 0.800 | 9 | 7 | 0.295 |
| Post influenza, no. | 0 | 1 | 1.000 | 6 | | 5 | 0.392 | 7 | 5 | 0.362 |
| Post Covid-19, no. | 2 | 2 | 0.590 | 7 | | 0 | 0.059 | 9 | 2 | 0.157 |
| Prior antifungal agent, no. | 13 | 8 | 0.269 | 10 | | 6 | 0.665 | 23 | 14 | 0.360 |
| Blood routine test | | | | | | | | | | |
| WBC, ×10^9^/L | 6.90 (5.56, 10.87) | 13.88 (7.72, 17.20) | 0.057 | 11.31 (7.72, 16.38) | | 9.75 (6.56, 15.78) | 0.710 | 8.97 (6.56, 15.16) | 11.93 (7.70, 15.91) | 0.366 |
| N, ×10^9^/L | 5.04 (4.04, 9.16) | 12.43 (5.12, 16.19) | 0.070 | 9.44 (5.10, 13.70) | | 8.42 (5.99, 13.38) | 0.793 | 6.64 (4.23, 10.83) | 10.68 (5.99, 13.82) | 0.152 |
| L, ×10^9^/L | 1.36±0.82 | 1.29±0.86 | 0.847 | 1.30±1.03 | | 0.76±0.44 | 0.015 | 1.32±0.95 | 0.92±0.63 | 0.073 |
| Hgb, g/L | 104.12±26.24 | 100.88±30.92 | 0.788 | 108.90±25.60 | | 106.81±24.63 | 0.789 | 107.21±25.66 | 104.83±26.36 | 0.715 |
| PLT, ×10^9^/L | 310.00 (229.00, 347.00) | 174.00 (78.00, 227.25) | 0.049 | 238.50 (120.75, 355.25) | | 177.50 (89.75, 255.75) | 0.362 | 245.00 (140.00, 351.00) | 177.50 (89.75, 250.75) | 0.063 |
| Inflammation-related test | | | | | | | | | | |
| ESR, mm/h | 27.00 (13.00, 45.00) | 37.00 (12.00, 64.50) | 0.733 | 32.50 (13.00, 65.75) | 43.50 (27.25, 60.75) | | 0.469 | 30.00 (13.00, 57.00) | 42.00 (25.75, 60.25) | 0.269 |
| CRP, mg/L | 5.39 (1.94, 10.08) | 6.66 (1.17, 21.20) | 0.951 | 8.00 (3.71, 14.33) | 20.40 (11.33, 28.78) | | 0.001 | 5.94 (3.17,12.40) | 16.40 (7.29, 28.10) | 0.008 |
| CD4+T cells, /μL | 377.50 (203.75, 687.50) | 247.00 (27.00, 627.50) | 0.383 | 341.00 (83.50, 522.00) | 113.00 (70.50, 267.00) | | 0.168 | 36.15 (105.50, 603.00) | 157.00 (47.75, 261.50) | 0.069 |
| Microbiological tests |  |  |  |  | |  |  |  |  |  |
| G test | 37.14±55.37 | 89.56±139.03 | 0.408 | 19.33 (10.00, 186.13) | | 137.20 (10.00, 462.40) | 0.244 | 18.38 (10.00, 141.18) | 41.82 (10.00, 268.67) | 0.237 |
| GM test (serum) | 0.26 (0.14, 0.62) | 0.17 (0.14, 0.74) | 0.535 | 0.49 (0.28, 1.28) | | 0.92 (0.27, 1.84) | 0.294 | 0.36 (0.22, 0.89) | 0.80 (0.17, 1.51) | 0.443 |
| GM test (balf) | - | - | - | 2.35 (0.42, 4.23) | | 4.25 (1.71, 5.35) | 0.127 | - | - | - |
| PCT, ng/mL | 0.10 (0.05, 1.59) | 0.95 (0.06, 1.58) | 0.425 | 0.12 (0.05, 1.50) | | 0.46 (0.14, 1.05) | 0.063 | 0.10 (0.05, 1.51) | 0.50 (0.13, 1.21) | 0.043 |
| Chest CT findings |  |  |  |  | |  |  |  |  |  |
| Consolidation, no. (%) | 15 | 6 | 0.413 | 19 | | 8 | 0.537 | 34 | 14 | 0.350 |
| Cavitation, no. (%) | 11 | 2 | 0.060 | 18 | | 5 | 0.102 | 29 | 7 | 0.016 |
| Multiple nodules, no. (%) | 10 | 1 | 0.022 | 10 | | 1 | 0.035 | 20 | 2 | 0.002 |
| Bronchial lumen stenosis, no. (%) | 7 | 2 | 0.424 | 3 | | 2 | 0.742 | 10 | 4 | 0.700 |
| Pleural effusion, no. (%) | 10 | 3 | 0.318 | 14 | | 6 | 0.679 | 24 | 9 | 0.355 |

| **Supplementary Table 2** Comparison of clinical characteristics, laboratory and chest radiology findings between ICU patients and non-ICU patients in PM and IPA. | | | | | | | | | | |
| --- | --- | --- | --- | --- | --- | --- | --- | --- | --- | --- |
|  | PM (n=25) | | | IPA (n=48) | | | | All patients (n=73) | | |
|  | ICU  (n=9) | Non-ICU  (n=16) | P value | ICU  (n=18) | | Non-ICU  (n=30) | P value | ICU  (n=27) | Non-ICU  (n=46) | P value |
| Ages, years, mean±SD | 49.67±15.45 | 54.31±12.91 | 0.429 | 55.61±13.52 | | 65.57±9.88 | 0.005 | 53.63±14.18 | 61.65±12.16 | 0.013 |
| Sex, male, no. (%) | 6 | 9 | 0.608 | 15 | | 24 | 0.773 | 21 | 33 | 0.570 |
| Underlying disease, no. (%) | | | | | | | | | | |
| DM | 7 | 13 | 0.836 | 7 | | 9 | 0.527 | 14 | 22 | 0.740 |
| DKA | 1 | 2 | 0.918 | 3 | | 0 | 0.047 | 4 | 2 | 0.124 |
| Chronic lung disease | 1 | 4 | 0.388 | 5 | | 7 | 0.731 | 6 | 11 | 0.869 |
| Solid organ cancer | - | - | - | 1 | | 3 | 0.579 | 1 | 3 | 0.600 |
| Hematologic disease | 0 | 1 | 1.000 | 2 | | 2 | 0.595 | 2 | 3 | 0.886 |
| Solid organ transplantation | 5 | 0 | 0.002 | 1 | | 1 | 0.713 | 6 | 1 | 0.005 |
| Autoimmune disease | - | - | - | 1 | | 4 | 0.373 | 1 | 4 | 0.394 |
| Hepatopathy | 2 | 1 | 0.249 | 2 | | 1 | 0.290 | 4 | 2 | 0.124 |
| Chronic kidney disease | 4 | 0 | 0.010 | 4 | | 2 | 0.121 | 8 | 2 | 0.003 |
| Systemic steroid, no. (%) | 7 | 6 | 0.048 | 10 | | 11 | 0.202 | 17 | 17 | 0.032 |
| Immunosuppressant, no. (%) | 5 | 1 | 0.005 | 3 | | 7 | 0.577 | 8 | 8 | 0.222 |
| Post influenza, no. (%) | 1 | 0 | 0.360 | 8 | | 4 | 0.017 | 9 | 4 | 0.009 |
| Post Covid-19, no. (%) | 2 | 2 | 0.531 | 3 | | 5 | 1.000 | 5 | 7 | 0.715 |
| Prior antifungal agent, no. (%) | 9 | 12 | 0.260 | 6 | | 10 | 1.000 | 15 | 22 | 0.524 |
| Blood routine test | | | | | | | | | | |
| WBC, ×10^9^/L | 12.99 (5.90, 21.09) | 7.56 (6.21, 11.82) | 0.251 | 13.02 (8.46, 16.39) | | 9.08 (6.84, 15.79) | 0.160 | 12.99 (7.62, 17.67) | 8.65 (6.41, 13.43) | 0.053 |
| N, ×10^9^/L | 12.63 (4.76, 20.40) | 5.15 (4.02, 9.95) | 0.073 | 11.38 (7.23, 14.68) | | 7.39 (4.15, 11.50) | 0.045 | 6.64 (4.23, 10.83) | 6.48 (4.06, 10.01) | 0.008 |
| L, ×10^9^/L | 0.87±0.78 | 1.59±0.73 | 0.041 | 0.93±0.63 | | 1.23±1.03 | 0.265 | 0.91±0.67 | 1.35±0.95 | 0.041 |
| Hgb, g/L | 101.56±35.22 | 103.94±22.82 | 0.858 | 104.78±26.63 | | 110.31±24.22 | 0.467 | 103.70±29.12 | 108.04±23.67 | 0.515 |
| PLT, ×10^9^/L | 140.00 (55.00, 291.00) | 308.00 (221.75, 349.00) | 0.065 | 135.00 (81.25, 248.25) | | 251.00 (168.75, 361.25) | 0.017 | 138.00 (70.00, 247.00) | 262.00 (189.25, 354.75) | 0.003 |
| Inflammation-related test | | | | | | | | | | |
| ESR, mm/h | 22.00 (13.00, 27.00) | 36.50 (13.00, 52.50) | 0.360 | 30.00 (10.00, 53.00) | 48.00 (25.00, 67.00) | | 0.112 | 26.50 (12.25, 40.25) | 43.00 (16.00, 65.00) | 0.081 |
| CRP, mg/L | 9.20 (3.00, 24.65) | 4.48 (1.73, 8.39) | 0.238 | 15.20 (7.24, 26.98) | 10.60 (3.78, 15.90) | | 0.048 | 12.25 (6.97, 26.00) | 5.67 (3.13, 13.83) | 0.016 |
| CD4+T cells, /μL | 60.00 15.00, 247.00) | 507.50 (296.00, 727.00) | 0.014 | 193.00 (73.50, 426.00) | 322.50 (82.50, 521.75) | | 0.534 | 162.00 (48.25, 321.50) | 377.50 (153.25, 622.75) | 0.034 |
| Microbiological tests |  |  |  |  | |  |  |  |  |  |
| G test | - | - | - | 186.16 (17.48, 462.50) | | 10.00 (10.00, 174.60) | 0.043 | 133.43 (10.00, 231.92) | 10.00 (10.00, 141.18) | 0.056 |
| GM test (serum) | 0.14 (0.12, 0.97) | 0.26 (0.17, 0.44) | 0.616 | 0.91 (0.27, 2.26) | | 0.49 (0.29, 1.32) | 0.273 | 0.76 (0.15, 1.41) | 0.36 (0.22, 0.86) | 0.431 |
| GM test (balf) | - | - | - | 4.04 (1.24, 5.45) | | 1.67 (0.27, 3.15) | 0.070 | - | - | - |
| PCT, ng/mL | 1.59 (1.07, 3.05) | 0.06 (0.05, 0.11) | 0.003 | 0.51 (0.09, 1.77) | | 0.16 (0.07, 0.47) | 0.093 | 1.21 (0.10, 2.07) | 0.12 (0.05, 0.35) | 0.001 |
| Chest CT findings |  |  |  |  | |  |  |  |  |  |
| Consolidation, no. (%) | 8 | 13 | 0.609 | 11 | | 16 | 0.599 | 19 | 29 | 0.524 |
| Cavitation, no. (%) | 3 | 10 | 0.158 | 7 | | 16 | 0.332 | 10 | 26 | 0.108 |
| Multiple nodules, no. (%) | 3 | 8 | 0.417 | 3 | | 8 | 0.417 | 6 | 16 | 0.259 |
| Bronchial lumen stenosis, no. (%) | 3 | 6 | 0.835 | 3 | | 2 | 0.281 | 6 | 8 | 0.613 |
| Pleural effusion, no. (%) | 6 | 7 | 0.267 | 9 | | 11 | 0.364 | 15 | 18 | 0.173 |

| **Supplementary Table 3** Comparison of clinical characteristics, laboratory and chest radiology findings between mechanically ventilated patient and non-mechanically ventilated patient in PM and IPA. | | | | | | | | | | |
| --- | --- | --- | --- | --- | --- | --- | --- | --- | --- | --- |
|  | PM (n=25) | | | IPA (n=48) | | | | All patients (n=73) | | |
|  | Mechanical ventilation (n=8) | Non- mechanical ventilation  (n=17) | P value | Mechanical ventilation (n=31) | | Non- mechanical ventilation  (n=17) | P value | Mechanical ventilation (n=25) | Non- mechanical ventilation  (n=47) | P value |
| Ages, years, mean±SD | 49.13±13.01 | 53.31±14.00 | 0.488 | 60.53±8.98 | | 62.55±13.81 | 0.590 | 56.88±11.52 | 59.40±14.42 | 0.452 |
| Sex, male, no. (%) | 5 | 10 | 1.000 | 16 | | 23 | 0.069 | 21 | 33 | 0.187 |
| Underlying disease, no. (%) | | | | | | | | | | |
| DM | 6 | 13 | 0.725 | 9 | | 7 | 0.033 | 15 | 20 | 0.158 |
| DKA | 2 | 1 | 0.205 | 3 | | 0 | 0.039 | 5 | 1 | 0.010 |
| Chronic lung disease | 0 | 4 | 0.262 | 4 | | 8 | 0.861 | 4 | 12 | 0.345 |
| Solid organ cancer | - | - | - | 1 | | 3 | 0.641 | 1 | 3 | 0.666 |
| Hematologic disease | 0 | 1 | 1.000 | 0 | | 4 | 0.282 | 0 | 5 | 0.156 |
| Solid organ transplantation | 4 | 1 | 0.014 | 0 | | 2 | 0.533 | 4 | 3 | 0.202 |
| Autoimmune disease | - | - | - | 1 | | 4 | 0.427 | 1 | 4 | 0.455 |
| Hepatopathy | 2 | 1 | 0.205 | 1 | | 2 | 0.938 | 3 | 3 | 0.422 |
| Chronic kidney disease | 3 | 1 | 0.059 | 2 | | 4 | 0.909 | 5 | 5 | 0.274 |
| Systemic steroid, no. (%) | 7 | 6 | 0.015 | 8 | | 13 | 0.732 | 15 | 19 | 0.113 |
| Immunosuppressant, no. (%) | 4 | 2 | 0.050 | 2 | | 8 | 0.235 | 6 | 10 | 0.791 |
| Post influenza, no. (%) | 1 | 0 | 0.333 | 7 | | 5 | 0.055 | 8 | 5 | 0.025 |
| Post Covid-19, no. (%) | 2 | 2 | 0.449 | 1 | | 7 | 0.112 | 3 | 9 | 0.428 |
| Prior antifungal agent, no. (%) | 8 | 12 | 0.262 | 6 | | 10 | 0.831 | 14 | 22 | 0.458 |
| Blood routine test | | | | | | | | | | |
| WBC, ×10^9^/L | 11.19 (6.22, 22.80) | 7.56 (6.21, 11.82) | 0.320 | 11.40 (8.28, 15.52) | | 11.16 (7.21, 16.70) | 0.821 | 11.40 (7.18, 15.84) | 8.76 (6.51, 14.80) | 0.318 |
| N, ×10^9^/L | 8.86 (4.68, 22.13) | 5.15 (3.22, 9.95) | 0.101 | 10.05 (7.04, 13.24) | | 7.71 (4.23, 14.00) | 0.348 | 10.05 (5.62, 13.71) | 6.58 (4.13, 11.38) | 0.079 |
| L, ×10^9^/L | 0.99±0.72 | 1.52±0.82 | 0.142 | 0.84±0.59 | | 1.27±1.02 | 0.112 | 0.89±0.62 | 1.35±0.96 | 0.031 |
| Hgb, g/L | 104.00±36.03 | 103.44±23.81 | 0.964 | 107.00±27.84 | | 108.87±23.75 | 0.809 | 106.04±29.97 | 106.98±23.65 | 0.893 |
| PLT, ×10^9^/L | 178.50 (58.50, 314.00) | 275.50 (219.00, 349.00) | 0.106 | 138.00 (77.50, 304.00) | | 244.00 (137.00, 343.00) | 0.106 | 140.00 (77.50, 297.00) | 245.00 (153.50, 347.00) | 0.031 |
| Inflammation-related test | | | | | | | | | | |
| ESR, mm/h | 17.50 (7.50, 26.25) | 36.50 (14.50, 52.50) | 0.062 | 32.50 (12.50, 52.50) | 39.00 (23.50, 67.50) | | 0.255 | 26.50 (9.75, 48.75) | 39.00 (15.75, 65.25) | 0.075 |
| CRP, mg/L | 7.29 (1.78, 25.80) | 5.16 (1.73, 8.45) | 0.535 | 17.20 (9.56, 27.65) | 9.19 (3.69, 15.40) | | 0.010 | 12.25 (7.29, 26.20) | 5.94 (3.14, 13.36) | 0.011 |
| CD4+T cells, /μL | 139.90 (39.00, 281.25) | 631.00 (251.00, 722.00) | 0.037 | 193.00 (98.00, 522.00) | 311.50 (78.25, 521.25) | | 0.678 | 193.00 (54.00, 340.00) | 352.00 (95.00, 625.50) | 0.075 |
| Microbiological tests |  |  |  |  | |  |  |  |  |  |
| G test | - | - | - | 133.43 (10.00, 462.50) | | 10.00 (10.00, 189.68) | 0.211 | 19.33 (10.00, 231.92) | 9.39 (10.00, 168.85) | 0.351 |
| GM test (serum) | 0.52 (0.14, 0.97) | 0.23 (0.11, 0.34) | 0.188 | 0.86 (0.26, 1.68) | | 0.50 (0.30, 1.50) | 0.696 | 0.75 (0.22, 1.19) | 0.35 (0.17, 0.92) | 0.298 |
| GM test (balf) | - | - | - | 3.93 (1.16, 4.61) | | 2.93 (0.46, 5.13) | 0.438 | - | - | - |
| PCT, ng/mL | 1.51 (0.33, 3.05) | 0.05 (0.02, 0.12) | 0.003 | 0.41 (0.12, 1.65) | | 0.12 (0.06, 0.79) | 0.044 | 0.61 (0.13, 2.17) | 0.10 (0.05, 0.39) | 0.001 |
| Chest CT findings |  |  |  |  | |  |  |  |  |  |
| Consolidation, no. (%) | 6 | 14 | 0.449 | 11 | | 16 | 0.382 | 17 | 30 | 0.723 |
| Cavitation, no. (%) | 3 | 10 | 0.245 | 6 | | 17 | 0.195 | 9 | 27 | 0.083 |
| Multiple nodules, no. (%) | 5 | 6 | 0.245 | 3 | | 8 | 0.514 | 8 | 14 | 0.846 |
| Bronchial lumen stenosis, no. (%) | 2 | 6 | 0.535 | 2 | | 3 | 0.822 | 4 | 9 | 0.739 |
| Pleural effusion, no. (%) | 4 | 8 | 1.000 | 7 | | 13 | 0.959 | 11 | 21 | 0.956 |

| **Supplementary Table 4** Predictive values of different assessment tools in different outcomes | | | | | |
| --- | --- | --- | --- | --- | --- |
| ICU stays | | | | | |
|  | AUC | 95%CI | P value | Sensitivity | Specificity |
| Post influenza + NLR | 0.779 | 0.665-0.869 | <0.001 | 69.23 | 82.22 |
| CURB-65 | 0.530 | 0.409-0.648 | 0.673 | 18.52 | 89.13 |
| CURB-65 + NLR | 0.723 | 0.604-0.823 | <0.001 | 53.85 | 82.22 |
| CURB-65 + NLR + Post influenza | 0.779 | 0.664-0.869 | <0.001 | 69.23 | 82.22 |
| SOFA | 0.704 | 0.585-0.805 | 0.001 | 59.26 | 69.57 |
| SOFA + NLR | 0.781 | 0.667-0.871 | <0.001 | 76.92 | 75.56 |
| SOFA + NLR + Post influenza | 0.830 | 0.722-0.909 | <0.001 | 84.62 | 71.11 |
| APACHE-Ⅱ | 0.715 | 0.597-0.814 | 0.001 | 62.96 | 71.74 |
| APACHE-Ⅱ+ NLR | 0.771 | 0.656-0.862 | <0.001 | 53.85 | 93.33 |
| APACHE-Ⅱ+ NLR + Post influenza | 0.844 | 0.738-0.919 | <0.001 | 76.92 | 82.22 |
| Mechanical ventilation | | | | | |
|  | AUC | 95%CI | P value | Sensitivity | Specificity |
| Post influenza + HbA1c | 0.709 | 0.577-0.819 | 0.005 | 72.73 | 71.05 |
| CURB-65 | 0.607 | 0.485-0.720 | 0.115 | 92.00 | 27.66 |
| CURB-65 + HbA1c | 0.698 | 0.566-0.810 | 0.005 | 54.55 | 78.95 |
| CURB-65 + HbA1c + Post influenza | 0.736 | 0.606-0.841 | 0.001 | 59.09 | 84.21 |
| SOFA | 0.718 | 0.599-0.818 | 0.001 | 84.00 | 55.32 |
| SOFA + HbA1c | 0.763 | 0.636-0.863 | <0.001 | 72.73 | 76.32 |
| SOFA + HbA1c + Post influenza | 0.772 | 0.646-0.870 | <0.001 | 68.18 | 81.58 |
| APACHE-Ⅱ | 0.690 | 0.570-0.794 | 0.003 | 68.00 | 63.83 |
| APACHE-Ⅱ + HbA1c | 0.745 | 0.616-0.849 | <0.001 | 59.09 | 84.21 |
| APACHE-Ⅱ+ HbA1c + Post influenza | 0.789 | 0.665-0.884 | <0.001 | 72.73 | 84.21 |
| 30-day mortality | | | |  |  |
|  | AUC | 95%CI | P value | Sensitivity | Specificity |
| CURB-65 | 0.702 | 0.584-0.804 | 0.001 | 100.00 | 30.61 |
| SOFA | 0.629 | 0.508-0.739 | 0.060 | 75.00 | 51.02 |
| APACHE-Ⅱ | 0.602 | 0.480-0.714 | 0.130 | 87.50 | 36.73 |
